# Isolation and Genotypic Characterization of *Toxoplasma gondii* Based on GRA6 Gene from Environmental Soil Samples in Mazandaran Province, North of Iran

**Published:** 2020

**Authors:** Beheshteh HAGHPARAST-KENARI, Shahabeddin SARVI, Mehdi SHARIF, Ehsan AHMADPOUR, Seyed Abdollah HOSSEINI, Ahmad DARYANI

**Affiliations:** 1. Student Research Committee, Mazandaran University of Medical Sciences, Sari, Iran; 2. Toxoplasmosis Research Center (TRC), Mazandaran University of Medical Sciences, Sari, Iran; 3. Department of Parasitology and Mycology, Sari Medical School, Mazandaran University of Medical Sciences, Sari, Iran; 4. Infectious and Tropical Diseases Research Center, Tabriz University of Medical Sciences, Tabriz, Iran

**Keywords:** *Toxoplasma gondii*, Soil, Genotype, GRA6 protein

## Abstract

**Background:**

Soil is one of the environmental sources of *Toxoplasma gondii* oocysts. The other hand, genotype of the parasite is one of the important factors for its pathogenicity. Due to the importance of toxoplasmosis on public health, this study aimed to isolation and genotyping of *T. gondii* in environmental soil samples of Mazandaran Province, north of Iran.

**Methods:**

Overall, 192 soil samples were collected from different areas in Mazandaran Province from Apr to Sep 2014. The flotation method was used for recovering oocysts. Then, soil samples were investigated for DNA detection of *T. gondii* using nested PCR of RE gene, genotyping with Semi-nested PCR of GRA6 gene and restriction fragment length polymorphism (RFLP) analysis. Results were analyzed using Chi-squared test. A significant difference was considered with a *P*<0.05.

**Results:**

From 192 soil samples, *T. gondii* DNA was detected in 150 samples (78.1%). Then genotype of 23 samples was determined (91.3% type I and 8.7% type II).

**Conclusion:**

Prevalence of *T. gondii* in soil samples of Mazandaran province, north of Iran is high and *T. gondii* GRA6 type I is predominant. Soil can be the most important source of severe toxoplasmosis in this province.

## Introduction

*Toxoplasma gondii* is an apicomplexan protozoan parasite and one of the most prevalent parasitic infections in humans and other warm-blooded creatures (1). Toxoplasmosis is often subclinical and asymptomatic in immunocompetent individuals but can be life-threatening in pregnant women and immunocompromised patients, for example, HIV-infected individuals, immunosuppressed and organ transplantation candidates. In AIDS patients, the most common clinical symptom of toxoplasmosis is focal encephalitis with headache, confusion and weakness. In pregnant women, it can lead to fetal diseases, including abortion, loss of vision, mental retardation, malformations and subclinical symptoms (2, 3). Moreover, neonatal deaths and abortion may be observed in animals infected with this parasite (4, 5). Animals and humans are infected with *T. gondii* through consumption of raw and undercooked infected meat and ingestion of mature oocysts from the environment, congenital, blood transfusion, organ transplantation (4, 5).

The pathogenesis of *T. gondii* in humans depends on parasite factors (strain virulence, inoculum size, and parasite stage in the life cycle) and host factors (immunity and genetic background) (6–8). *T. gondii* has been classified into three main genotypes including I, II, III and some atypical genotypes (9, 10). Type I isolates cause lethal toxoplasmosis in all strains of laboratory mice, whereas type II and III isolates are significantly less virulent (11).

About 15% to 85% of people in the world (12), 39.3% of the general population in Iran and 54% of individuals in north of Iran (near the Caspian Sea) are infected with *T. gondii* infection (13). Mazandaran Province has the highest seroprevalence rate of *T. gondii* in Iran. It may be due to suitable climate conditions that can cause survival of oocysts in soil (14).

Cats are the definitive hosts of *T. gondii*. Soil contamination with *T. gondii* oocysts is related to distribution of infected cat feces in environment. Gardens, parks and around rubbish dump are main places that cats may excrete feces in soil (15).

Sporulated oocysts can survive for 18 months in soil at different temperatures and 28 d in freezing conditions. They are resistant to chemical and physical agents (4, 16, 17). Moreover, oocysts survive in damp conditions more than dry conditions (18). Contact with contaminated soil by *T. gondii* oocyst can be the cause of toxoplasmosis outbreaks (4, 17). So far, several soil-born transmission toxoplasmosis outbreaks have been recorded in the USA and Brazil (19, 20). Some studies were performed to survey *T. gondii* oocysts in soil samples by molecular and mouse bioassay methods and *T. gondii* parasite was found in 5%–37.9% of soil samples (9, 20–25).

Due to the public health importance of toxoplasmosis and lack of *T. gondii* genetic information, the present study was performed for molecular detection and genetic characterizations of *T. gondii* in environmental soil samples in Mazandaran province, north of Iran.

## Materials and Methods

### Sampling

Overall, 192 soil samples were collected from Apr to Sep 2014 from rural and urban areas in six cities of Mazandaran province (north of Iran). This province geographically was divided into two regions, east and west. Ninety-six samples were collected from east areas (Behshahr, Sari, Savadkooh cities) and 96 samples were from west areas (Fereidoonkenar, Chalous, Ramsar cities). The samples were taken from different sites where the population of cats or their feces often were high, such as areas around rubbish dumps, parks, public places, vegetable gardens, sand heaps, forest and shadow areas near houses. For each soil sample, 300–500 g of the soil was collected from 2–5 cm ground depth of 5–10 different parts of each site and then was transported to the laboratory in clean bags. Soil samples were dried at laboratory temperature for 1 to 2 d, and sifted through a 100 mesh sieve (26, 27). Finally, 7 g of the prepared soil was used for further examinations.

### Recovery of oocysts

Concentration of oocysts was performed by sucrose solution floatation method as described previously with some modification (27). Briefly, 7 g of the prepared soil mixed in 50 ml of 0.1% Tween-80 solution and gelatin 0.1% and then centrifuged at 1100 g for 10 min. Next, the supernatant was removed and sediment mixed in 5 ml of sucrose solution with gelatin 0.1% and centrifuged at 1100 g for 10 min. Then the supernatant was transferred to another tube, and after dilution with distilled water (1:10), centrifuged as described above. Then the sediment was transferred to 1.5 ml microtube and stored at −20 °C for further examination.

### DNA Extraction

After 3 freeze-thaw cycles in liquid nitrogen alternated with a 95 °C water bath, DNA extraction was performed with the commercial Denazist genomic DNA isolation kit III (Mashhad, Iran), according to manufacturer’s instructions. After that, DNA was stored in −20 °C for further examinations.

### Detection of Toxoplasma gondii parasite

We used RE gene marker that has 200–300 copies in *T. gondii* genome as the target for nested-PCR.

A pair of outer primers, REF1-5′-TGACTCGGGCCCAGCTGCGT-3′, RER1-5′-CTCCTCCCTTCGTCCAAGCCTCC-3′, a pair of inner primers, REF2-5′-AGGGACAGAAGTCGAAGGGG-3′, RER2-5′-GCAGCCAAGCCGGAAACATC-3′ (AF146527) (28) were used for nested-PCR reaction. The first round of PCR reaction mixture contained of 12.5 μl of Taq 2x Master Mix (Ampliqon, Denmark), 10 pmole of each primer, 5 μl of template DNA and to remove PCR inhibitor 2 μl of 1% BSA was added in a final volume of 25 μl. PCR protocol was performed according to study conducted earlier (29) except annealing at 60 °C. Sterile distilled water and DNA of *T. gondii* tachyzoites RH-strain was used as a PCR negative and positive control, respectively. In the second round of PCR, reaction mixture contained 12.5 μl of Taq 2x Master Mix (Ampliqon, Denmark), 10 pmole of each primer, 2 μl of first-round PCR product in a final volume of 25 μl. PCR protocol was performed (29) except annealing at 60 °C. C1000^TM^ Thermal cycler (BIO-RAD) was used for amplification. In addition, for confirming negative samples, all of the negative samples were checked by adding 1 μl of genomic DNA from *T. gondii* tachyzoites of RH-strain to DNA template of negative soil samples for rejecting or confirming PCR inhibitors. Finally, PCR products were analyzed after electrophoresis on 1.5% agarose gel stained with SYBR Green using Gel Doc System (UVITEC Cambridge).

### Sequencing of detected T. gondii parasite

In order to confirm results, randomly two positive nested-PCR products were sequenced by Macrogen Lab (Korea).

### Genotyping of detected Toxoplasma gondii parasite

Genetic characterization was determined by RFLP on products of semi nested-PCR using GRA6 gene, a single copy gene marker of *Toxoplasma gondii* parasite.

A pair of outer primer, GRA6F1-5′- GGCAAACAAAACGAAGTG -3′, GRA6R1-5′- TCGCCGAAGAGTTGACATAG -3′, and a pair of inner primer, GRA6F2-5′- TTTCCGAGCAGGTGACCT -3′, GRA6R2-5′- TCGCCGAAGAGTTGACATAG -3 (similar to GRA6R1) (30, 31) were used for semi nested-PCR reaction. The first round of PCR reaction mixture contained of 12.5 μl of Taq 2x Master Mix (Ampliqon, Denmark), 10 pmole of each primer, 5 μl of template DNA and 2 μl of BSA 1% was added in a final volume of 25 μl. Amplification was performed using the following conditions: one cycle of 7 min initial denaturation at 95 °C followed by 35 cycles of 95 °C for 40 sec, 59 °C for 35 sec, 72 °C for 1 min, and was ended by 1 cycle of final extension at 72 °C for 5 min. Sterile distilled water and DNA of *T. gondii* tachyzoites (RH-strain) were used as negative and positive control, respectively. In the second round of PCR, reaction mixture contained 12.5 μl of Taq 2x Master Mix (Ampliqon, Denmark), 10 pmole of each primer, 1 μl of first-round PCR product in a final volume of 25 μl. Amplification was performed using the following conditions: one cycle of 5 min initial denaturation at 95 °C followed by 40 cycles of 95 °C for 40 sec, 57 °C for 30 sec, 70 °C for 30 sec, and was ended by 1 cycle of final extension at 72 °C for 7 min. Finally, PCR products were analyzed after electrophoresis on 2% agarose gel stained with SYBR Green using Gel Doc System (UVITEC cambridge). Some of the final semi nested-PCR products were purified using Accuprep Gel purification Kit (BIONEER) according to the manufacturer's method.

To identify *T. gondii* genotypes, the restriction analysis method was performed using MseI enzyme (Fermentas, Germany) according to the manufacture’s instruction. Analysis of digested products was done after electrophoresis on 2% agarose gel stained with SYBR Green using Gel Doc System (UVITEC Cambridge).

### Sequencing of genotyped T. gondii parasite

Two PCR products (*GRA6* genes) were purified using Accuprep Gel purification Kit (Cat.No.K-3035-1) (BIONEER) according to manufacturer’s method and were sequenced by Macrogen Lab (Korea).

### Statistical analysis

Statistical analysis was done using SPSS ver. 16 (Chicago, IL, USA). Results were analyzed using Chi-squared test. A significant difference was considered with a *P*-value<0.05.

## Results

### Detection of Toxoplasma gondii by nested PCR

**Fig. 1:**
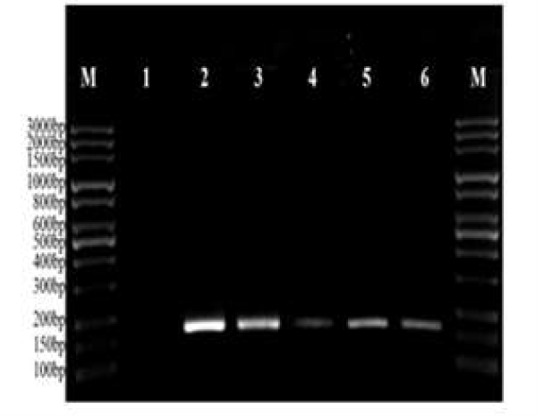
Gel image of RE amplification products of *T. gondii*. Lane M, molecular weight marker 100 bp (Jena Bioscience); Lane 1, negative control; Lane 2, positive control; Lane3–6, positive samples (PCR amplified a 164 bp fragment of RE gene marker)

From 192 soil samples, *Toxoplasma gondii* DNA was detected in 150 samples (78.1%) by nested-PCR on RE gene. All of negative samples were negative for the presence PCR inhibitor. The length of fragment was 164 bp from RE gen (AF146527) (Fig. 1).

The contamination of soil samples with *T. gondii* in East of Mazandaran (81.2%) was more than West of Mazandaran with 75% of contamination, and the highest and lowest contamination of *T. gondii* were observed in Behshar (87.5%) and Chalous city (68.8%), the respectively (Table 1). However, statistically, no significant difference was observed between the existence of *T. gondii* DNA in soil samples of different cities (*X^2^* =6.217, *P*=0.286). The contamination of rural areas with 80.4% was more than urban areas with 77.4% contamination (Table 1). Statistically, it was not significant (*x^2^*=0.189, *P*=0.664).

**Table 1:** The frequency rate of *T. gondii* DNA in soil samples in north of Iran by cities and area in 2014

***Area***	***City***	***Number of samples***	***No. Positive samples by Nested - PCR (%)***	**P*-value***
Cities	Behshahr	32	28 (87.5)	0.286
	Sari	32	27 (84.3)	
	Savadkooh	32	23 (71.8)	
	Fereidoonkenar	32	23 (71.8)	
	Chalous	32	22 (68.8)	
	Ramsar	32	27 (84.3)	
	Total	192	150 (78.1)	
Area	Urban	146	113 (77.4)	0.664
	Rural	46	37 (80.4)	
	Total	192	150 (78.1)	

Seasonal study of *T. gondii* in soil samples of Mazandaran showed that contamination in the summer season (79.2%) was more than spring (77.1%) (Table 2). However, statistically, no significant difference was observed between the existence of *T. gondii* DNA in soil samples in different seasons (*x^2^*=0.122, *P*=0.727).

**Table 2:** The frequency rate of *T. gondii* DNA in soil samples of Mazandaran, north of Iran by seasons

***Season***	***Number of samples***	***No. Positive samples by nested-PCR (%)***	**P*-value***
Spring	96	74 (77)	0.727
Summer	96	76 (79.2)	
Total	192	150 (78.1)	

Overall, all of location of sampling were contaminated with *T. gondii* and the highest and the lowest contamination was observed in vegetable garden (94.1%) and public places (73%), respectively (Table 3). Statistically, no significant difference was observed among the existence of *T. gondii* DNA in different types of soil samples (*x^2^*=3.576, *P*=0.734).

**Table 3:** The frequency rate of *Toxoplasma gondii* in Mazandaran province, north of Iran by different types of soil

***Location of sampling***	***Number of samples***	***No. positive samples by nested-PCR (%)***	**P*-value***
Park	61	47 (77)	0.734
Vegetable garden	17	16 (94.1)	
Shadow areas near houses	41	31 (75.6)	
Public places	37	27 (73)	
Around of rubbish dump	19	15 (79)	
Sand heap	10	8 (80)	
Forest	7	6 (85.7)	
Total	192	150 (78.1)	

### Genotype characterization of detected T. gondii parasite by semi nested PCR

**Fig. 2:**
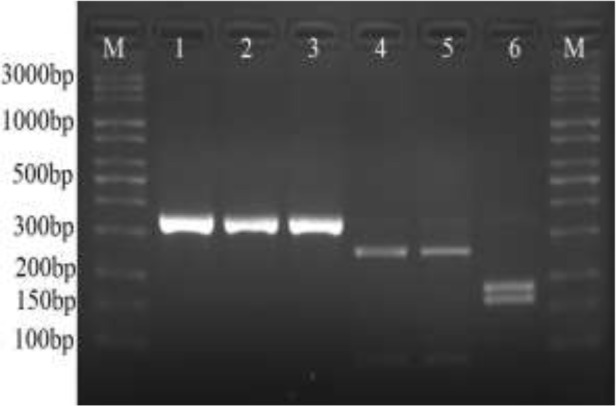
Gel image of GRA6 amplification products of *T. gondii* (Lane 1–3) and MseI digestions of *Toxoplasma gondii* GRA6 amplification products (lane 4–6). Lane M, molecular weight marker 100 bp (Jena Bioscience); lane 1, positive control (RH strain); lane 2–3, positive soil samples; lane 4, positive control (RH strain); Lane 5, GRA6 type I parasite of soil samples; lane 6, GRA6 type II parasite of soil samples

Only 23 of 150 (15.3%) *T. gondii* positive soil samples with nested-PCR on RE gen were amplified with semi nested-PCR on GRA6 gen. The length of fragment was 344 bp from GRA6 gen. RFLP analysis determined that 21/23 soil samples (91.3%) were type I (The length of fragments were 258 bp and 86 bp) and 2/23 soil samples (8.7%) were type II (The length of fragments were 183 bp and 161 bp) (Fig. 2).

### Sequencing of detected T. gondii

Nucleotide sequence data of *T. gondii* obtained in present study have been deposited in the Genbank and provided the accession numbers KX231335, KX231336 for GRA6 gene and KU873096, KU873097 for RE gene.

## Discussion

Soil is one of the environmental sources of *T. gondii* oocysts. These oocysts can remain infective for months to years. Studying the distribution of soil contamination by *T. gondii* will be useful in understanding the risk of toxoplasmosis in both intermediate hosts and humans (15, 32).

Unfortunately, evaluation of *T. gondii* oocysts contamination in environment has been limited by lack of reliable detection methods. So far, little information has been gathered on the presence of oocysts in natural environment, especially in soil. The present study is the first investigation of *T. gondii* on environmental soil contamination in this area using molecular detection methods.

Molecular methods are necessary to recover low numbers of oocysts and to discriminate *T. gondii* from closely related coccidian, for example, *Hamondia, Neoaspora and Besnoitia*. We used RE gene marker for molecular detection of *T. gondii* that has 200–300 copies in *T. gondii* genome as the target for nested-PCR (21, 22).

This study is important to understand the situation of *T. gondii* in soils of rural and urban areas in north of Iran, Mazandaran. Prevalence of toxoplasmosis among Iranian general population is 39.3% (13). However, this infection in different groups of Mazandaran’s people is very high. For example, 75.6% in healthy people, 77.4% in AIDS/HIV patients (33) and 58.8% in pregnant women (10). Moreover, seroprevalence of toxoplasmosis was observed 22% among schoolchildren in north of Iran, Sari city that it was positively related to contact with soil (34). Our study estimated that the prevalence rate of this parasite in soil is 78.1% that was more than other studies (9, 20–25).

The geographical location and weather conditions as temperature and humidity are effective for survive of oocysts. Toxoplasmosis is more prevalent in humid areas in comparison to dry areas. The infection risk increases when the weather is both warm and humid, or moderated and less humid (35). The high prevalence of *T. gondii* in soil of northern Iran could be due to many factors, including the humidity up to approximately 90%, environmental temperature 18–20 °C and existence of a lot of cats.

Prevalence rate of *T. gondii* in soil samples was 17.8% in Poland. It used NANO_3_ saturated solution flotation, DNA extraction with commercial kit, molecular method with conventional PCR, based on RE and B1 genes that were able to detect at least 10^3^ *T. gondii* oocysts in 40 g of soil (22). In Tehran, Iran, the prevalence rate of *T. gondii* in soil samples was reported 8.7% (9) that method was similar to Lass and colleagues' method. The prevalence rate of coccidian oocysts in soil samples using sucrose flotation method (42%) was found more than NANO3 saturated solution flotation method (27%) (36).

Prevalence rate of *T. gondii* in soil samples found 16.27% using conventional PCR based on B1 and RE genes and 23.02% by LAMP method based on MIC3 gene. Directly 0.5 gr soil was used, without flotation for concentration of oocysts and amount of used soil was lower than other studies. Sensitivity of conventional PCR based on RE gene and LAMP-based on MIC3 was determined 5 *T. gondii* tachyzoites in soil. Besides, sensitivity of conventional PCR based on B1 gene was determined 50 *T. gondii* tachyzoites. Prevalence of *T. gondii* in soil has been decreased from spring to winter season (21). While in most cases, there are not seasonal variations of oocysts (4). In addition, in our study was not found significant difference between two seasons with prevalence of *T. gondii.*

Prevalence of *T. gondii* in soil samples of pig farms in central china, was reported 21.1% using conventional PCR method based on RE and B1 genes and 37.9% by LAMP method based on MIC3 gene (23). Contamination of soil samples with *T. gondii* was observed 5% in Arak city in Iran using molecular method (25) and 22.58% in Brazil by mouse bioassay method (24). In a study, oocysts detection limit was reported 25 oocysts in 30 g of soil or 1 oocyst in 1 gram of soil. They added 0.1% gelatin to the washing and floating solution for removing interference soil's factors (such as humic acid) to recovery of oocysts. The number of recovered oocysts increased in comparison with gelatin-free solutions. Moreover, combination of modified sucrose flotation method with gelatin and PCR inhibitor removal procedure could remove the technical problems in detection of *T. gondii* oocysts in soil (27). Since the sensitivity of Matsuo et al method is higher than Lass et al method, we used Matsuo et al method to detect *T. gondii* in soil. In our study, we reported higher *T. gondii* contamination in soil samples compared with other studies. It must be due to using DNA extraction kit, performance of nested-PCR based on RE gene and collecting soil samples around cat feces or where cats population were high. However, prevalence of *T. gondii* DNA in soil samples of Mazandaran Province, northern Iran is high that could be an important source of contamination for humans and animals.

*T. gondii* parasite has been classified into three main genotypes including I, II, III and some atypical genotypes (9, 10). Multilocus-PCR-RFLP genotyping of 11 gene markers (SAG1, SAG2, alt-SAG2, SAG3, BTUB, GRA6, C22-8, L358, C29-2, PK1, Apico) among approximately 1500 samples worldwide has revealed 189 different genotypes of *T. gondii.*

The data were shown in *Toxoplasma* database (ToxoDB) (http://www.toxodb.org/toxo/).

Type I or type I variants of *T. gondii* are responsible for severe toxoplasmic retinochoroiditis, and the atypical genotype causes acute toxoplasmosis in immunocompetent individuals.

Type I isolates cause lethal toxoplasmosis in all strains of laboratory mice, whereas type II and III isolates are significantly less virulent (11).

There is a little information about genotype transmission patterns in Mazandaran Province.

In a study on animals and humans, type II and III were identified with Multiplex PCR method (using microsatellite markers) in Iran [Mazandaran and Tehran] (7).

In the present study, of 150 soil samples, only 23 samples (15.3%) were positive for the GRA6 gene in PCR, while 78.1% of the samples were positive for the RE gene in PCR. This difference in PCR results is due to be single copy of the GRA6 gene and conversely, high copy of the RE gene repeated about 200 to 300 times in the entire genome of *T. gondii*. Therefore, it is certain that the percentage of positive cases in GRA6 in PCR is much lower than that of the RE.

In our study, we could detect GRA6 type I (21 samples, 91.3%) and II (2 samples, 8.7%) in soil samples and these results were unexpected because type I was unusual in animals and humans in Iran (Mazandaran and Tehran).

In this study, nucleotide sequence analysis of two sequenced products confirmed that our isolates belonged to type I and type II of *T. gondii* which both of the sequences are available in Genbank with accession numbers: KX231335 (type I), KX231336 (type II).

Habibi et al identified type I from sheep aborted fetal samples in Qazvin Province, Iran (37). In southwest of Iran isolated type II and type III from birds (38). Our genotyping results were similar to study of Lass et al that found SAG 2 type I (5 samples) and SAG2 type II (2 samples) in soil samples of Poland (22) and were different with study of Tavalla et al, that identified SAG 2 type III (9 samples), SAG 2 type I (1 sample) and mix type I and III (3 samples) in soil samples of Tehran, Iran (9).

One of the limitations of this study was that it was bettr to carry out the nucleotide sequencing on all isolates. Moreover, more gene markers were used to determine genotypes.

## Conclusion

The frequency rate of *T. gondii* in soil samples of north of Iran is high and *T. gondii* GRA6 type I is predominant in Mazandaran’s soil samples. Soil can be the most important source of severe toxoplasmosis in this province. For understanding of transmission patterns, further studies on genetic characterization of *T. gondii* in soil, humans and animals using multilocus-PCR in this area are recommended.
